# Investigating the Antioxidant and Cytocompatibility of *Mimusops elengi* Linn Extract over Human Gingival Fibroblast Cells

**DOI:** 10.3390/ijerph18137162

**Published:** 2021-07-04

**Authors:** Shaeesta Khaleelahmed Bhavikatti, Mohmed Isaqali Karobari, Siti Lailatul Akmar Zainuddin, Anand Marya, Sameer J. Nadaf, Vijay J. Sawant, Sandeep B. Patil, Adith Venugopal, Pietro Messina, Giuseppe Alessandro Scardina

**Affiliations:** 1 Department of Periodontics, School of Dental Sciences, Universiti Sains Malaysia, Health Campus, Kubang Kerian 16150, Malaysia; lailatul@usm.my or; 2 Division of Periodontics and Community Dental Sciences, College of Dentistry, King Khalid University, Abha 62529, Saudi Arabia; 3 Conservative Dentistry Unit, School of Dental Sciences, Universiti Sains Malaysia, Health Campus, Kubang Kerian 16150, Malaysia; dr.isaq@gmail.com; 4 Department of Conservative Dentistry & Endodontics, Saveetha Dental College & Hospitals, Saveetha Institute of Medical and Technical Sciences University, Chennai 600077, India; 5 Department of Orthodontics, Faculty of Dentistry University of Puthisastra, Phnom Penh 12211, Cambodia; amarya@puthisastra.edu.kh; 6 Department of Orthodontics, Saveetha Institute of Medical and Technical Sciences, Saveetha Dental College, Saveetha University, Chennai 600077, India; avenugopal@puthisastra.edu.kh; 7 Sant Gajanan Maharaj College of Pharmacy, Mahagaon 416503, India; sam.nadaf@rediffmail.com; 8 Department of Chemistry, Smt. K. W. College, Sangli 416416, India; sawantvjkwc@gmail.com; 9 Department of Pharmacology, Dr. Shivajirao Kadam College of Pharmacy, Kasbe digraj, Sangli 416305, India; sandeeppharmacology@gmail.com; 10 Department of Surgical Oncological and Stomatological Disciplines, University of Palermo, 90133 Palermo, Italy; pietro.messina01@unipa.it

**Keywords:** chlorhexidine, cytotoxicity, fibroblast, gingival, herbs, medicinal

## Abstract

Background—chlorhexidine (CHX) is most commonly used as a chemical plaque control agent. Nevertheless, its adverse effects, including teeth discoloration, taste alteration and calculus build-up, limit its use and divert us to medicinal herbs. The purpose of the study was to evaluate the phytochemical composition, antioxidant potential, and cytotoxic effects of *Mimusops elengi Linn* extract (ME) over normal human cultured adult gingival fibroblasts (HGFs). Methods—in vitro phytochemical screening, total flavonoid content, antioxidant potential by DPPH and Nitric Oxide (NO) radical scavenging activity, and cytotoxic effects of ME extracts over HGF were explored. The viability of HGF cells was determined using 3-(4,5-dimethylthiazol-2-yl)-2, 5-diphenyl tetrazolium bromide (MTT), neutral red uptake, and trypan blue assay after treatment with different concentrations of CHX and ME (0.3125 to 10 µg/mL). Results**—**ME showed some alkaloids, glycosides, saponins and flavonoids exhibited relatively moderate-to-good antioxidant potential. Increasing the concentration of CHX and ME from 0.3125 to 10 µg/mL reduced cell viability from 29.71% to 1.07% and 96.12% to 56.02%, respectively. At higher concentrations, CHX reduced the viability of cells by 52.36-fold compared to ME, revealed by MTT assay. At 10 µg/mL concentration, the mean cell viability of CHX and ME-treated cells was 2.24% and 57.45%, respectively, revealed by a neutral red assay. The viability of CHX- and ME-treated HGF cells estimated at higher concentrations (10 µg/mL) using trypan blue assay was found to be 2.18% and 47.36%, respectively. A paired *t*-test showed significance (*p* < 0.05), and one-way ANOVA difference between the mean cell viability of CHX- and ME-treated cells at different concentrations. One-way ANOVA confirmed the significant difference between the viability of CHX- and ME-treated cells. Conclusions—The cytoprotective and antioxidant effects of ME emphasize its potential benefits. Therefore, it could emerge as a herbal alternative and adjunct to conventional oral hygiene methods, that can diminish periodontal tissue destruction.

## 1. Introduction

A dental plaque is a structurally and functionally organized biofilm of diverse microbial composition [1]. Accumulation of dental plaque is ultimately known to result in caries, gingivitis, and periodontal diseases [2]. Periodontitis is a common oral inflammatory disease, multifactorial in its etiology and associated with the destruction of the periodontium and the tissues supporting the tooth. The primary cause for such periodontal destruction is oral bacteria that eventually result in tooth loss [3]. There is a plethora of evidence implicating reactive oxygen species (ROS), derived predominantly from polymorphonuclear leukocytes, in the pathogenesis of periodontal tissue destruction. These ROS cause tissue damage through an array of different mechanisms, such as DNA damage, lipid peroxidation, protein disruption and stimulation of inflammatory cytokine release [4,5,6]. Therefore, combating this oxidative stress using safe, economic medications and having the least or no adverse effects could possibly assist periodontal disease management.

Chemical plaque control measures are used as adjuvants to conventional mechanical methods and are known to interfere with biofilm composition and metabolism [3]. Among the mouthwashes used for chemical plaque control, chlorhexidine gluconate (CHX) is the most used antiplaque agent and is considered the “gold standard” owing to its broad-spectrum antimicrobial activity against different oral pathogens such as *Streptococcus mutans*, *Streptococcus oralis*, *Lactobacillus acidophilus*, *Lactobacillus fermentum*, *Candida albicans,* etc. [7,8]. However, specific side effects like tooth staining, taste disturbance, calculus build-up, etc., have limited its applications [7,9]. Very recently, a study by Polizzi et al. (2019) revealed that CHX 0.12% with alcohol and CHX 0.20% with alcohol mouthwashes showed a significant presence of extrinsic tooth staining [10]. The human gingival fibroblasts (HGFs) are essential periodontal connective tissue cells that aid in wound healing. Cytotoxic effects of the chemical plaque control agents over the HGFs are a matter of concern. There is documented evidence of the cytotoxic effects of CHX over the HGFs. Coelho et al. (2020) observed that exposure of the fibroblasts to the mouthwashes caused a G2/M phase block and cell death predominantly by necrosis [11]. This directs towards the use of chemical plaque control agents with cytoprotective effects.

Several natural plant-based products are widely used for diverse therapeutic applications owing to their safe and potential medicinal properties [12,13]. Recently available studies offer essential data that herbal products may comprise akin antimicrobial potential to reputable chemotherapeutics. The World Health Organization guidelines define herbal medicines as finished, labeled medicinal products containing an active ingredient, i.e., obtained from the aerial or underground parts of botanicals or other plant materials or their combination [14]. Specifically, since ancient days, *Mimusops elengi* Lin, a wild plant distributed in tropical and subtropical regions belonging to the family Sapotaceae, has been known for its myriad of medicinal values [15]. So far, various parts of the plant have been used in traditional medicine to manage pain, inflammation, wounds and so on [16]. It has several known benefits such as anti-bacterial, antiviral, anti-inflammatory, antihyperglycemic, antioxidant, etc. [17]. Chewing of the root bark strengthens the teeth and escalates oral health [18]. In Ayurveda, *M. elengi* has been reported to be used for arresting bleeding gums [19]. The use of unripe fruit and seed for fixing loose teeth is documented [20]. Herbal mouth rinse derived from *M. elengi* bark aqueous extract acts as a potent plaque inhibitor and anti-inflammatory agent in gingivitis [21]. Chloroform extract of *M. elengi* bark exhibited prominent anti-bacterial activity in dental patients by the ditch plate technique [22]. Whereas, ethanolic extracts of bark, leaves and seeds *M. elengi* are reported to be anti-bacterial agents against some pathogens [23].

Notably, for administrating agents, along with the antimicrobial activity against the oral pathogens, selective cytotoxicity towards bacteria with diminished toxic effects to host cells is also equally essential [24,25,26]. Though studies are reporting the beneficial properties of this herb against oral pathogens, there is a paucity of literature on its cytocompatibility. In comparison with previous reports, our study investigated cytocompatibility of *M. elengi* extract against HGF cells and related it with CHX using three different cytotoxicity assays. Further, the present investigation included potential antioxidant determination of the aforementioned extract, which gives insights into the mechanism. Collectively, this leads to the hypothesis that ME may have lesser toxic effects on HGF cells than CHX.

Hence, the primary objectives of this study were to screen the phytochemical composition, antioxidant capacities and determine and compare the cytocompatibility activity of ME with CHX (available as Rexidin 0.2%) over HGFs.

## 2. Materials and Methods

### 2.1. Study Protocol

An in vitro experimental design was adopted to perform the study. The study was registered in the Scientific Research Committee, College of Dentistry, King Khalid University (SRC/REG/2018-2019/91). The study was conducted in full accordance with the declared ethical principles (World Medical Association Declaration of Helsinki, version VII, 2013. Ethical clearance was obtained from the institution’s independent ethics committee (Approval No. SRC/ETH/2018-19/116).

### 2.2. Collection of Plant Material and Processing of the Extract

The bark of *Mimusops elengi* Linn was collected from the surrounding regions of Maharashtra, India. The plant material was identified and authenticated by a botanist. A voucher specimen of each plant was deposited in the department. Plant materials were washed with tap water and dried in an oven at 45 °C for seven days. The material was ground; the fine powder was made and stored in an air-tight container until use. The coarse powder was packed in a Soxhlet apparatus and continuously extracted with petroleum ether at temperature 100–120 °C till all fat constituents were separated out and then extracted with ethanol at temperature 60–80 °C till all the constituents were separated. For a powder weight of 100 gms, the extractive value was 13%.

### 2.3. Phytochemical Screening

Qualitative phytochemical screening was performed to check the presence of alkaloids, sterols, glycosides, flavonoids, tannins, proteins, as per standard protocol (Table 1).

### 2.4. Evaluation of Total Flavanoid Content

**Figure 1:**
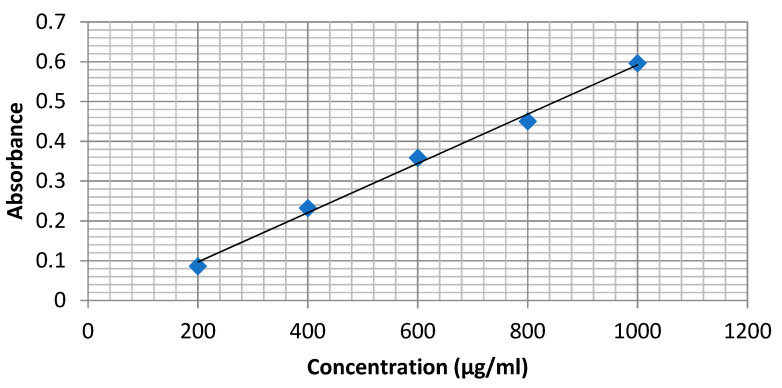
Calibration curve of Quercetin.

The aluminum chloride colorimetric assay measured total flavonoid content. Briefly, the reaction mixture containing 1 mL of ethanolic extract and 4 mL of distilled water was prepared in a 10 mL volumetric flask and to which 0.30 mL of 5% sodium nitrite was added. After 5 min, 0.3 mL of 10% aluminum chloride was added and mixed. Then, 2 mL of 1 M sodium hydroxide was treated and diluted to 10 mL with distilled water. A similar set of reference standard solutions of quercetin (200, 400, 600, 800 and 1000 μg/mL) was prepared. The absorbance for test and standard solutions was determined against the reagent blank at 510 nm using a spectrophotometer. The total flavonoid content was expressed as mg of quercetin equivalents (QE) per g of extract. The absorbance of the test sample was performed in triplicate (Figure 1).

### 2.5. Quantification of Antioxidant Activities

#### 2.5.1. DPPH Free Radical Scavenging Assay

Molecule 1, 1-diphenyl-2-picrylhydrazyl (a a-diphenyl-bpicrylhydrazyl; DPPH) is characterized as a stable free radical by virtue of the delocalization of the spare electron over the molecule as a whole so that the molecule does not dimerize, as would be the case with most other free radicals. The delocalization of electrons also gives rise to the deep violet color, characterized by an absorption band in ethanol solution centered at about 517 nm. When a solution of DPPH is mixed with that of a substrate (AH) that can donate a hydrogen atom, this gives rise to the reduced form with the loss of this violet color.

The ability of compounds to scavenge the DPPH radical was assessed using the previously reported method [27,28] with few modifications. Briefly, 1 mL of herbal extract (200, 400, 600, 800, and 1000 µg/mL) was mixed with 3.0 mL DPPH (0.5 mmol/L in methanol), and the resultant absorbance was recorded at 517 nm after 30 min incubation at 37 °C. The percentage of scavenging activity was derived using the following formula,

$$\mathrm{Percentage}\;\mathrm{of}\;\mathrm{inhibition}\;\left(\%\right)=\left(\frac{A\;\mathrm{control}-A\;\mathrm{sample}}{A\;\mathrm{control}}\right)\times 100$$

where A control—absorbance of DPPH, A sample—absorbance reaction mixture (DPPH with Sample).

#### 2.5.2. Nitric Oxide Radical Scavenging Activity 

NO· is generated in biological tissues by specific nitric oxide synthases, which metabolize arginine to citrulline with the formation of NO· via a five-electron oxidative reaction [29]. The compound sodium nitroprusside is known to decompose in an aqueous solution at physiological pH (7.2), producing NO·. Under aerobic conditions, NO· reacts with oxygen to produce stable products (nitrate and nitrite), the quantities of which can be determined using Griess reagent [30].

A total of 1 mL of 10 mM sodium nitroprusside dissolved in 0.5 mL phosphate buffer saline (pH 7.4) was mixed with 1 mL of 1 mM synthetic compounds in DMSO. The mixture was incubated at 25 °C for 150 min. After incubation, the reaction mixture was mixed with 1.0 mL of pre-prepared Griess reagent (1.0 mL sulfanilic acid reagent (0.33% in 20% glacial acetic acid at room temperature for 5 min with 1 mL of naphthylethylenediamine dichloride (0.1% *w*/*v*)). The mixture was then incubated at room temperature for 30 min and its absorbance poured into a cuvette was measured at 546 nm. The decreasing absorbance indicates a high nitric oxide scavenging activity.

The amount of nitric oxide radical inhibition was calculated following this equation:

$$\%\;\mathrm{inhibition}\;\mathrm{of}\;\mathrm{NO}\;\mathrm{radical}=\left(\frac{A_{0}-{\;A}_{1}}{A_{0}}\right)\times 100$$

where A_0_ is the absorbance before reaction and A_1_ is the absorbance after the reaction has taken place with the Griess reagent.

### 2.6. Cytotoxic Activity

#### 2.6.1. Materials

Dulbecco’s Modified Eagle Media (DMEM) and Fetal bovine serum (FBS) with low glucose were purchased from Gibco, Invitrogen. Antimycotic 100× solution was procured from Thermofisher Scientific. Neutral Red GRM122 and Trypan blue TC193 were procured from Hi-Media, Mumbai.

#### 2.6.2. Cell Culture

After obtaining informed consent, normal human adult primary gingival fibroblast (HGF) cells were obtained from healthy gingival tissue of a human adult premolar that was excised during periodontal surgery. These cells were cultured in Dulbecco’s Modified Eagle Media (DMEM medium). Further, it was supplemented with 10% fetal calf serum (FBS) and 1% Antibiotic-Antimycotic 100× solution followed by incubation in a CO_2_ incubator (Eppendorf, New Brunswick, Galaxy 170 R, Germany) maintained at 37 °C, 5% CO_2_ with 95% humidity until the completion of experiments [31].

#### 2.6.3. MTT Assay

The cells were seeded in a 96-well flat-bottom microplate and maintained at 37 ºC in 95% humidity and 5% CO_2_ overnight. Different concentrations (10%, 5%, 2.5%, 1.25%, 0.625%, 0.312% *w*/*v*) of samples were treated. The cells were incubated for another 48 h. The wells were washed twice with Phosphate Buffered Saline (PBS), and 20 μL of the MTT staining solution was added to each well, and the plate was incubated at 37 ºC. After 4 h, 100 μL of dimethyl sulfoxide (DMSO) was added to each well to dissolve the formazan crystals, and optical density (OD) was recorded with a 570 nm using a microplate reader [32].

Formula:

$$\mathrm{Surviving}\;\mathrm{cells}\;\left(\%\right)=\left(\frac{\mathrm{Mean}\;\mathrm{OD}\;\mathrm{of}\;\mathrm{test}\;\mathrm{compound}}{\mathrm{Mean}\;\mathrm{OD}\;\mathrm{of}\;\mathrm{Negative}\;\mathrm{control}\;}\right)\times 100$$

#### 2.6.4. Neutral Red Uptake Assay

Approximately 5 × 10^4^ cells per well were plated in 96-well plates, and DMEM containing 5% FBS was added and allowed to attach overnight. Different concentrations (10%, 5%, 2.5%, 1.25%, 0.625%, 0.312% *w*/*v*) of samples were treated. The cells were incubated for another 48 h. The wells were washed twice with PBS. A total of 100 mL of neutral red medium was added to each well of the plate. The plate was incubated for 2 h at the appropriate culture conditions. The neutral red medium was removed, and the wells were washed with PBS. A total of 150 mL neutral red de-stain solution was then added per well. The plate was shaken rapidly on a microtiter plate shaker for at least 10 min, or until the neutral red had been extracted from the cells and had formed a homogeneous solution. The OD of the neutral red extract was measured at 540 nm in a microtiter plate reader spectrophotometer, using blanks that contain no cells as a reference [33].

#### 2.6.5. Trypan Blue Assay

Approximately 5 × 10^4^cells per well were plated in 96-well plates, and DMEM containing 5% FBS was added and allowed to attach overnight. Different concentrations (10%, 5%, 2.5%, 1.25%, 0.625%, 0.312% *v*/*v*) of samples were treated. The cells were incubated for another 48 h. The wells were washed twice with PBS. The cell suspension was centrifuged for about 1500 rpm for 3 min, and the supernatant was discarded. The cell pellets were re-suspended in 1 mL PBS or serum-free complete medium. The 0.4% trypan blue and cell suspension were mixed. The mixture was incubated for 3 min at room temperature. A drop of the Trypan blue/cell mixture was applied to a hemacytometer. The hemacytometer was placed on the stage of a binocular microscope, and the focus was on the cells. The unstained (viable) and stained (nonviable) cells were counted separately in the hemacytometer. To obtain the total number of viable cells per ml of aliquot, the total number of viable cells was multiplied by the dilution factor for trypan blue. To obtain the total number of cells per ml of aliquot, the total number of viable and nonviable cells was added [34]. The percentage of viable cells was calculated as follows:

$$\mathrm{Viable}\;\mathrm{cells}\;\left(\%\right)=\frac{\mathrm{Total}\;\mathrm{number}\;\mathrm{of}\;\mathrm{viable}\;\mathrm{cells}}{\;\mathrm{total}\;\mathrm{number}\;\mathrm{of}\;\mathrm{cells}}\times 100$$

### 2.7. Statistical Analysis

The normality of data was estimated using the Kolmogorov–Smirnov test and the Shapiro–Wilk test. Statistical difference was tested using one-way analysis of variance (ANOVA) followed by post-hoc Tukey HSD Test, Scheffé, Bonferroni and Holm multiple comparisons. To ensure the reliability of ME-treated HGF cell viability estimated using MTT, neutral red and Trypan blue assay, data were compared with each other using ANOVA. The same analysis was performed on the results of CHX-treated HGF cell viability.

## 3. Results

### 3.1. Phytochemical Analysis

The results after phytochemical analysis are as depicted in Table 1.

### 3.2. Total Flavanoid Content

Total flavonoid contents were observed to be 867.52 ± 6.53 (μg QE/g). The calibration curve of quercetin is shown in Figure 1.

### 3.3. Antioxidant Activities

ME showed a concentration-dependent antioxidant effect against free radicals generated, revealed by DPPH and NO free radical scavenging assay. ME exhibited good activity compared with the standard drug when tested using DPPH assay, whereas it demonstrated moderate activity after testing using the NO radical scavenging assay. Detailed results are shown in Table 2.

### 3.4. Cytotoxicity Assay’s 

#### 3.4.1. MTT Assay

**Figure 2:**
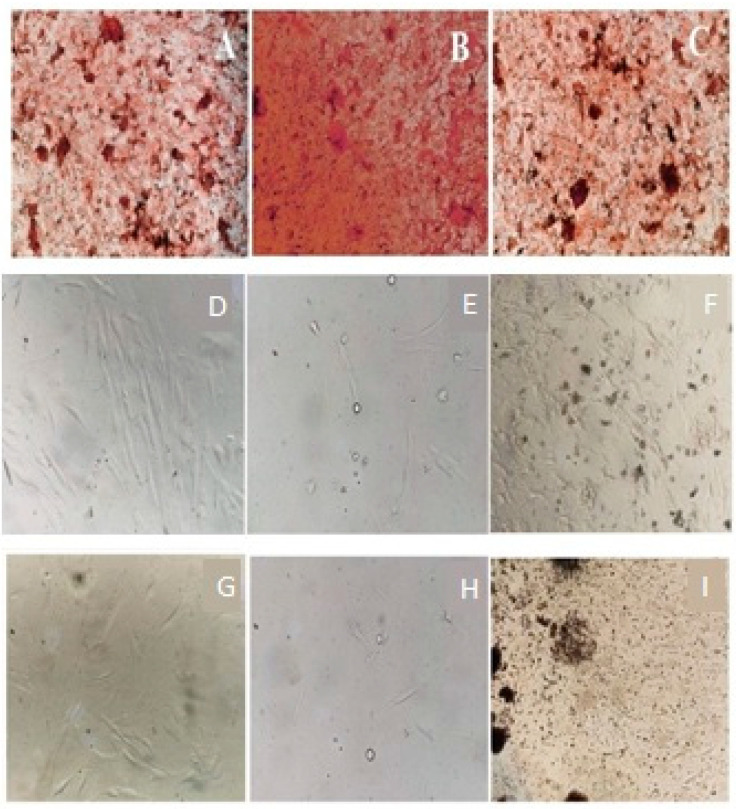
Viability study of (**A**,**D**,**G**)—negative control; (**B**,**E**,**H**)—CHX treated; and (**C**,**F**,**I**)—ME-treated cells using MTT assay (**A**–**C**), Neutral red uptake assay (**D**–**F**) and Trypan blue assay (**G**–**I**), respectively.

Both CHX and ME inhibited the proliferation of HGF cells but to a different extent in a dose-dependent matter. Increasing the concentration of CHX and ME from 0.3125 to 10 µg/mL reduced cell viability from 29.71% to 1.07% and 96.12% to 56.02%, respectively. At higher concentrations, CHX reduced the viability of cells by 52.36-fold. The paired *t*-test showed a significant (*p* < 0.05) difference between the mean cell viability of CHK- and ME-treated cells at different concentrations. Kolmogorov–Smirnov test and Shapiro–Wilk test exhibited the normality of the data. Lower D (<0.2), higher *p* (>0.9), skewness and kurtosis (close to zero) confirmed the normal distribution. One-way ANOVA showed a significant difference (F = 65.59949; *p* = 0.000011) between the viability of CHX- and ME-treated HGF cells estimated using MTT assay. Post-hoc Tukey HSD Test, Scheffé, Bonferroni and Holm multiple comparisons also confirmed the statistical significance (*p* < 0.01). Detailed results are shown in Figure 2A–C and Table 3, Table 4, Table 5, Table 6 and Table 7.

#### 3.4.2. Neutral Red Uptake Assay

CHX and ME reduced cell viability to a different extent. CHX and PH showed toxicity to approximately 98% and 42% of cells, respectively. CHX showed 2.3-fold higher toxicity to HGF cells compared to ME. The paired *t*-test showed a significant (*p* < 0.05) difference between the mean cell viability of CHX- and ME-treated cells at different concentrations. Lower D (<0.2), higher *p* (>0.9), and skewness, kurtosis values confirmed the normal distribution, revealed by the Kolmogorov–Smirnov test. One-way ANOVA showed a significant difference (F = 56.4817; *p* = 0.00002) between the viability of CHX- and ME-treated HGF cells estimated using the neutral red assay. This is also supported by the post-hoc Tukey HSD test, Scheffé, Bonferroni and Holm multiple comparisons (*p* < 0.01). Cells treated with CHX and ME were analyzed using the neutral red assay. Detailed results are shown in Figure 2D–F and Table 3, Table 4, Table 5, Table 6 and Table 7.

#### 3.4.3. Trypan Blue Assay

At higher concentrations (10 µg/mL), CHX exhibited toxicity to almost 98% of HGF cells, whereas ME reduced the proliferation of approximately 53% of cells. Notably, CHX showed 1.85-fold higher toxicity to HGF cells. Increasing the concentration of CHX and ME from 0.3125 to 10 µg/mL reduced cell viability from 25.58% to 2.1% and 92.64% to 47.36%, respectively (Table 2). The paired *t*-test showed a significant (*p* < 0.05) difference between the mean cell viability of CHX- and ME-treated cells at different concentrations. The Kolmogorov–Smirnov test and Shapiro–Wick test confirmed the normality of the data and hence were analyzed using ANOVA. The value of F (53.71799) and *p* (0.000025) confirmed statistical significance within the CHX- and ME-treated cells. The descriptive statistics of ANOVA are shown in Table 3. Post-hoc Tukey HSD test, Scheffé, Bonferroni and Holm multiple comparisons (*p* < 0.01) supported ANOVA results. Cells treated with CHX and ME were analyzed using trypan blue assay.

Notably, ANOVA showed that the HGF cells’ mean viability after performing the MTT, neutral red, and trypan blue assay was not significantly different (*p* < 0.05). This confirms the reliability of results obtained using three assays. Likewise, the viability results of CHK-treated cells were not significantly different. Descriptive statistics of ANOVA, Post-hoc Tukey HSD test, Scheffé, Bonferroni and Holm multiple comparisons have also been made. Detailed results are shown in Figure 2G–I and Table 3, Table 4, Table 5, Table 6 and Table 7.

## 4. Discussion

Periodontal diseases are one of the most prevalent chronic conditions globally. Periodontal disease is initiated by the colonization of bacterial pathogens, such as *Porphyromonasgingivalis*, *AggregatibacteractinomycetemcomitansTannerellaforsythus* and *T. denticola*. These microorganisms can stimulate the host defense mechanisms to produce reactive oxygen species that damage the nearby host tissue in addition to destroying the pathogens [35]. The lethal action of neutrophil-derived radicals is resisted by the antioxidant enzyme possessed by *p. gingivalis*. The ramifications of this process result in host tissue damage that may subsequently contribute to the destructive process in periodontal disease [36,37]. Therefore, efficient medications having potent antioxidant activities with the least or no adverse effects are needed.

Researchers have conjectured that 0.12% and 0.2% CHX with alcohol exhibits significant extrinsic tooth discoloration [38]. Owing to these adverse effects of CHX, various other materials, including nanomaterials, have been studied in dentistry [39,40], and nanoparticle-based antiplaque agents have shown to be efficient in oral biofilm reduction [41]. Presently, an increasingly large variety of phytoconstituents are being studied in experimental models to accomplish the knowledge of their biologic activity in vitro and in vivo [42]. Findings from the present phytochemical analysis revealed that *Mimusops elengi* Linn (bark) contains active ingredients like alkaloids, sterol, triterpenoids, glycosides, Anthraquinone glycosides, Saponins, Carbohydrates, Flavonoids, Tannins, and proteins. These compounds are known to have anti-bacterial, anti-inflammatory, and antifungal properties [43]. The higher total flavonoid content as estimated in the present study can be associated with the good antioxidant activities reported.

In the present study, cell viability was determined by 3-(4,5-dimethylthiazol-2-yl)-2, 5-diphenyl tetrazolium bromide (MTT assay) that measures the mitochondrial activity of live cells [44], neutral red uptake assay, where lysosomal uptake of neutral red dye is a highly sensitive indicator of cell viability [45] and Trypan blue assay, which is based on the principle that living cells possess intact cell membranes that exclude this dye, whereas dead cells do not [34]. All three assays showed cell viability. These in vitro tests using cell culture are beneficial due to their simplicity, quickness, low costs, and control of some experimental conditions (pH, CO_2_ concentration, and levels of some molecules) [46,47]. All three assays showed cell viability and based on the results obtained, the null hypothesis is rejected, and the alternate hypothesis stating that there is a significant difference between viability of ME and CHX treated cells is accepted. The present study results emphasize that low concentrations of the herbal product and CHX produced lesser cytotoxic effects. However, the herbal product significantly retained the cell viability of CHX, revealing its cytoprotective ability (Table 2). This is consistent with the previously published reports [47,48]. This study showed considerable modulation capability (inhibition) depending on the concentration of the product, which is consistent with the findings of [49].

In the present study, the high values of cell viability at lower concentrations may be attributed to the presence of some phytochemical components, such as glucose molecules from tannins and flavonoids. However, the cytotoxicity at higher concentrations may be attributed to the polyphenolic compounds in the herbal product [38,43]. There is a dearth of literature on the cytotoxic effects of ME on human gingival fibroblasts. However, the results of a study by [50] revealed a significant decrease in percent mitotic index and root length with time and increasing concentration when cytotoxic effects of ethanolic extract of bark of ME were investigated on meristematic cells of root tips of *Allium cepa* [50,51] in their study reporting the toxic effect of CHX beyond 1% on human gingival fibroblasts at 1-, 5-, and 15-min time exposure. However, *Azadirachta indica* extract did not adversely affect the fibroblasts even up to 50% concentration, revealing a less toxic effect than CHX on the cells [51].

Clinical studies would be beneficial to extrapolate the situation under intact in vivo conditions, where fibroblast interaction with other cellular components including the vasculature is crucial. This is one of the limitations of *in vitro* studies as in the present study and warrants a need for clinical studies to mimic the *in vitro* results obtained. Also a larger sample size of studies is needed to validate the cytoprotective potential of the medicinal herb.

## 5. Conclusions

Within the limitations of the study, it revealed that, the *Mimusops elengi* Linn bark extract exhibited moderate-to-good antioxidant potential and lowered the cytotoxic effects against HGF cells compared to CHX. This leads us to conclude that it can be used without damage to the gingival tissues. This improved activity could be attributed to its antioxidant potential. The findings of this study highlight the need for further evaluation of the product in clinical studies for its practical and safe application in dental plaque control.

## Figures and Tables

**Table 1:** Active ingredients in the ME as evaluated.

Chemical Constituents	Name of Test	Observed Changes	Result
Alkaloids	Mayer’s Reagent	White-colored turbidity	+
	Wagner’s Reagent	Reddish Brown Precipitate	+
	Hager’s Reagent	Yellow Precipitate floating	+
	Ehrlich’s Reagent	Two separate yellow and brown colored layers	+
Sterols &Triterpenoids	Salkowaski test	The lower layer turns red	+
	Sulphur test	Sinks in it	+
Glycosides	Baljet’s test	Yellow to orange color.	+
	Keller killani test	No Separation between two layers, lower layer reddish-brown and upper layer turns bluish-green	+
Anthraquinone glycosides	Borntrager’s test	The ammonical layer turns pink or red.	+
Saponins	Foam test	Formation of foam	+
Carbohydrates	Molisch’s test:		ND
	Barfoed’s test		ND
	Benedict’s test	Reddish-brown precipitate	+
Flavonoids	Shinoda test	Pink to magenta-red color	+
	Alkaline reagent test	Yellow color becomes a color lesson addition of few drops of dilute acid	+
	Lead acetate solution test	Yellow precipitate	+
Tannins	Ferric-chloride test	Dark color	+
Proteins	Millon’s test		ND
	Xanthoproteic test	No Yellow precipitate	_
	Biuret test	No Blue color	_
	Ninhydrin test	No Blue color.	_

Note: +, indicates the presence of phytoconstituents; _, indicates an absence of phytoconstituents; ND, indicates not determined.

**Table 2:** Antioxidant activities of *Mimusops elengi* Linn extract (DPPH and NO assays).

**DPPH ASSAY**		
**Sample**	**Absorbance at 517 nm**	**% inhibition**
**Control**	0.34	
**Standard Ascorbic acid (1 mg/mL)**	0.04	88.23
200 µg/mL	0.14	58.82
400 µg/mL	0.10	70.58
600 µg/mL	0.08	76.47
800 µg/mL	0.07	79.41
1000 µg/mL	0.05	85.29
**NO ASSAY**		
**Sample**	**Absorbance at 546 nm**	**% inhibition**
**Control**	1.64	
**Standard Ascorbic acid (1 mg/mL)**	0.28	82.92
200 µg/mL	1.49	08.87
400 µg/mL	1.25	23.78
600 µg/mL	1.00	39.02
800 µg/mL	0.77	53.04
1000 µg/mL	0.74	54.87

**Table 3:** Cell viability of Primary Gingival Fibroblasts determined using MTT, neutral red, and Trypan blue assay.

Mean Cell Viability % (Primary Gingival Fibroblast)						
Concentration (µg/mL)	MTT Assay		Neutral Red Assay		Trypan Blue Assay	
	CHX	ME	CHX	ME	CHX	ME
10	1.07	56.02	2.24	57.45	2.18	47.36
5	5.94	65.40	6.31	66.00	4.58	58.46
2.5	11.11	75.87	12.63	76.54	13.01	73.08
1.25	17.12	84.94	18.24	87.42	18.60	80.45
0.625	24.69	92.94	30.50	93.68	21.21	86.99
0.3125	29.71	96.12	33.64	97.46	25.58	92.64
Negative Control	100		100		100	

**Table 4:** Descriptive ANOVA statistics.

Source	Sum of Squares ss	Degrees of Freedom	Mean Squarems	F Statistic	*p*-Value
**MTT assay**					
Treatment	12,138.0602	1	12,138.0602	65.5995	** 0.000011
Error	1850.3285	10	185.0328		
Total	13,988.3887	11			
**Neutral red assay**					
Treatment	11,718.1250	1	11,718.1250	56.4817	** 0.00002
Error	2074.6763	10	207.4676		
Total	13,792.8013	11			
**Trypan blue assay**					
Treatment	1941.7979	1	10,432.4244	53.7256	** 0.000025
Error	12,374.2223	10	194.1798		
Total	1941.7979	11			

** indicated statistical significance.

**Table 5:** Descriptive statistics of multiple comparisons using a statistical test.

Post-hoc Tukey HSD Test						
Assay	Treatments pair	Tukey HSD Q statistic	Tukey HSD *p*-value		Tukey HSD inference	
MTT	CHX vs. ME	11.4542	0.0010053		** *p* < 0.01	
NR		10.6284	0.0010053		** *p* < 0.01	
TB		10.3659	0.0010053		** *p* < 0.01	
**Scheffé Multiple Comparison**						
Assay	Treatments pair	Scheffé TT-statistic	Scheffé *p*-value		Scheffé inference	
MTT	CHX vs. ME	8.0994	1.0567 × 10^−5^		** *p* < 0.01	
NR		7.5154	2.0266 × 10^−5^		** *p* < 0.01	
TB		7.3298	2.5123 × 10^−5^		** *p* < 0.01	
**Bonferroni and Holm Multiple Comparisons**						
Assay	Treatments pair	Bonferroni and Holm TT-statistic	Bonferroni *p*-value	Bonferroni inference	Holm *p*-value	Holm inference
MTT	CHX vs. ME	8.0994	1.0567 × 10^−5^	** *p* < 0.01	1.0567 × 10^−5^	** *p* < 0.01
NR		7.5154	2.0266 × 10^−5^	** *p* < 0.01	2.0266 × 10^−5^	** *p* < 0.01
TB		7.3298	2.5123 × 10^−5^	** *p* < 0.01	2.5123 × 10^−5^	** *p* < 0.01

NR: Neutral red; TB: Trypan Blue. ** indicate significant results.

**Table 6:** Descriptive statistics of ANOVA considering the comparison of viability CHK- and PH-treated HGF cells estimated using a different assay.

Description	Source	Sum of Squares ss	Degrees of Freedom	Mean Squarems	F Statistic	*p*-Value
ME treated cells	treatment	147.9926	2	73.9963	0.2766	* 0.7622
	error	4013.1105	15	267.5407		
	total	4161.1030	17			
CHX treated cells	treatment	30.6475	2	15.3237	0.1240	* 0.8843
	error	1853.8300	15	123.5887		
	total	1884.4775	17			

* indicates non-significant results.

**Table 7:** Descriptive statistics demonstrating comparisons of viability results obtained using different assays.

Post-hoc Tukey HSD Test					
Treatment pairs		Tukey HSD Q statistic	Tukey HSD *p*-value	Tukey HSD inference	
ME treated cells					
A vs. B		0.1812	0.8999947	insignificant	
A vs. C		0.8067	0.8266214	insignificant	
B vs. C		0.9879	0.7545422	insignificant	
CHX treated cells					
A vs. B		0.5112	0.8999947	insignificant	
A vs. C		0.1639	0.8999947	insignificant	
B vs. C		0.6751	0.8789620	insignificant	
**Scheffé Multiple Comparisons**					
Treatments pair		Scheffé TT-statistic	Scheffé *p*-value	Scheffé inference	
ME treated cells					
A vs. B		0.1281	0.9918294	insignificant	
A vs. C		0.5704	0.8513402	insignificant	
B vs. C		0.6985	0.7865543	insignificant	
CHX treated cells					
A vs. B		0.3615	0.9370267	insignificant	
A vs. C		0.1159	0.9933079	insignificant	
B vs. C		0.4774	0.8930757	insignificant	
**Bonferroni and Holm Multiple Comparisons**					
Treatments pair	Bonferroni and Holm TT-statistic	Bonferroni *p*-value	Bonferroni inference	Holm *p*-value	Holm inference
ME treated cells					
A vs. B	0.1281	2.6992443	insignificant	0.8997481	insignificant
A vs. C	0.5704	1.7305385	insignificant	1.1536923	insignificant
B vs. C	0.6985	1.4865869	insignificant	1.4865869	insignificant
CHK treated cells					
A vs. B	0.3615	2.1683911	insignificant	1.4455941	insignificant
A vs. C	0.1159	2.7277837	insignificant	0.9092612	insignificant
B vs. C	0.4774	1.9199406	insignificant	1.9199406	insignificant

A: MTT assay; B; Neutral red assay; C: Trypan blue assay.

## Data Availability

All data used to support the findings of this study are included in the article.
